# Associations of Serum and Red Blood Cell Folate With All-Cause and Cardiovascular Mortality Among Hypertensive Patients With Elevated Homocysteine

**DOI:** 10.3389/fnut.2022.849561

**Published:** 2022-02-25

**Authors:** Long Zhou, Hui Huang, Xiaoxiao Wen, Yu Chen, Jie Liao, Fuli Chen, Liancheng Zhao, Mingjiang Liu, Jianhong Tao, Gang Li

**Affiliations:** ^1^Department of Cardiology, Sichuan Provincial People's Hospital, University of Electronic Science and Technology of China, Chengdu, China; ^2^Department of Epidemiology, College of Public Health and Health Professions and College of Medicine, University of Florida, Gainesville, FL, United States; ^3^Division of Prevention and Community Health, National Center for Cardiovascular Disease, Fuwai Hospital, Chinese Academy of Medical Sciences and Peking Union Medical College, Beijing, China

**Keywords:** serum folate, red blood cell, hypertension, homocysteine, mortality

## Abstract

**Objectives:**

This study aims to explore the associations between serum and red blood cell (RBC) folate as indicators of short- and long-term folate status, respectively, and all-cause as well as CVD mortality among hypertensive patients with elevated homocysteine.

**Methods:**

A prospective cohort study of the National Health and Nutrition Examination Survey (1999–2006) and 2015 Linked Mortality File was performed. All-cause and CVD mortality risk estimated using Cox proportional hazards models with adjusting for multiple potential covariates.

**Results:**

A total of 1,753 hypertensive patients with elevated homocysteine [mean (SD) age, 68.5 (13.1)] were included in the analysis. During a median follow-up of 10.0 years, a total of 899 all-cause and 257 CVD deaths occurred. Compared the highest with the lowest quartile of RBC folate, the multivariable adjusted hazard ratios and 95% confidence intervals for all-cause and CVD death were 1.13 (0.92–1.39) and 1.47 (1.01–2.16) respectively. There was a significant and positive trend between RBC folate and the risk of CVD death (*p* for trend = 0.0196). No significant association was found between serum folate and mortality risk among the study sample.

**Conclusions:**

High level of RBC folate is associated with an increased risk of cardiovascular mortality among hypertensive patients with elevated homocysteine while serum folate has no such effects.

## Introduction

Hypertension or high blood pressure (BP) is one of the leading modifiable risk factors for cardiovascular disease (CVD) and all-cause mortality worldwide (1, 2). It was estimated that the number of people aged 30–79 years with hypertension doubled from 1990 to 2019, from 331 million women and 317 million men in 1990 to 626 million women and 652 million men in 2019 (3). Both genetic and prospective cohort studies yield strong evidence that the association between homocysteine and CVD is causal, wherein lowering serum homocysteine concentrations by 3 μmol/L was shown to reduce the risk of ischemic heart disease by 16% and stroke by 24% (4). More importantly, hypertension and elevated homocysteine levels may enhance the ability to provoke the risk of CVD and all-cause mortality each other (5). Previous studies showed that subjects with both elevated homocysteine (≥10 μmol/L) and hypertension (referred to as “H-type hypertension”) have a significantly higher risk of stroke compared with those without either condition (6, 7). Folate is naturally present in a wide variety of foods, including vegetables (especially dark green leafy vegetables), fruits and fruit juices, nuts, beans, peas, seafood, eggs, dairy products, meat, poultry, and grains. Spinach, liver, asparagus, and brussels sprouts are among the foods with the highest folate levels (8). Folate is critically involved in homocysteine metabolism. Elevated homocysteine is usually closely linked to inadequate folate intake or status (7, 9). For these reasons, folic acid supplementation has been recommended for hypertensive patients, especially those with elevated homocysteine, as a primary prevention strategy of stroke (10). However, our previous analysis showed that the use of folic acid supplements could significantly increase red blood cell (RBC) folate concentrations, which were further associated with an increased risk of severe abdominal aortic calcification (11). In contrast to serum folate which reflects recent intakes, RBC folate reflects both the folate stored in the liver and the long-term average consumption of folate over the life span of RBCs (12). As an extension to our previous work in identifying the potential adverse health outcomes of high folate levels, this study aimed to determine the associations of serum and RBC folate with all-cause and cardiovascular mortality among hypertensive patients with elevated homocysteine using the National Health and Nutrition Examination Survey (NHANES), 1999–2006 and linked mortality data.

## Methods

### Study Population

**Figure 1:**
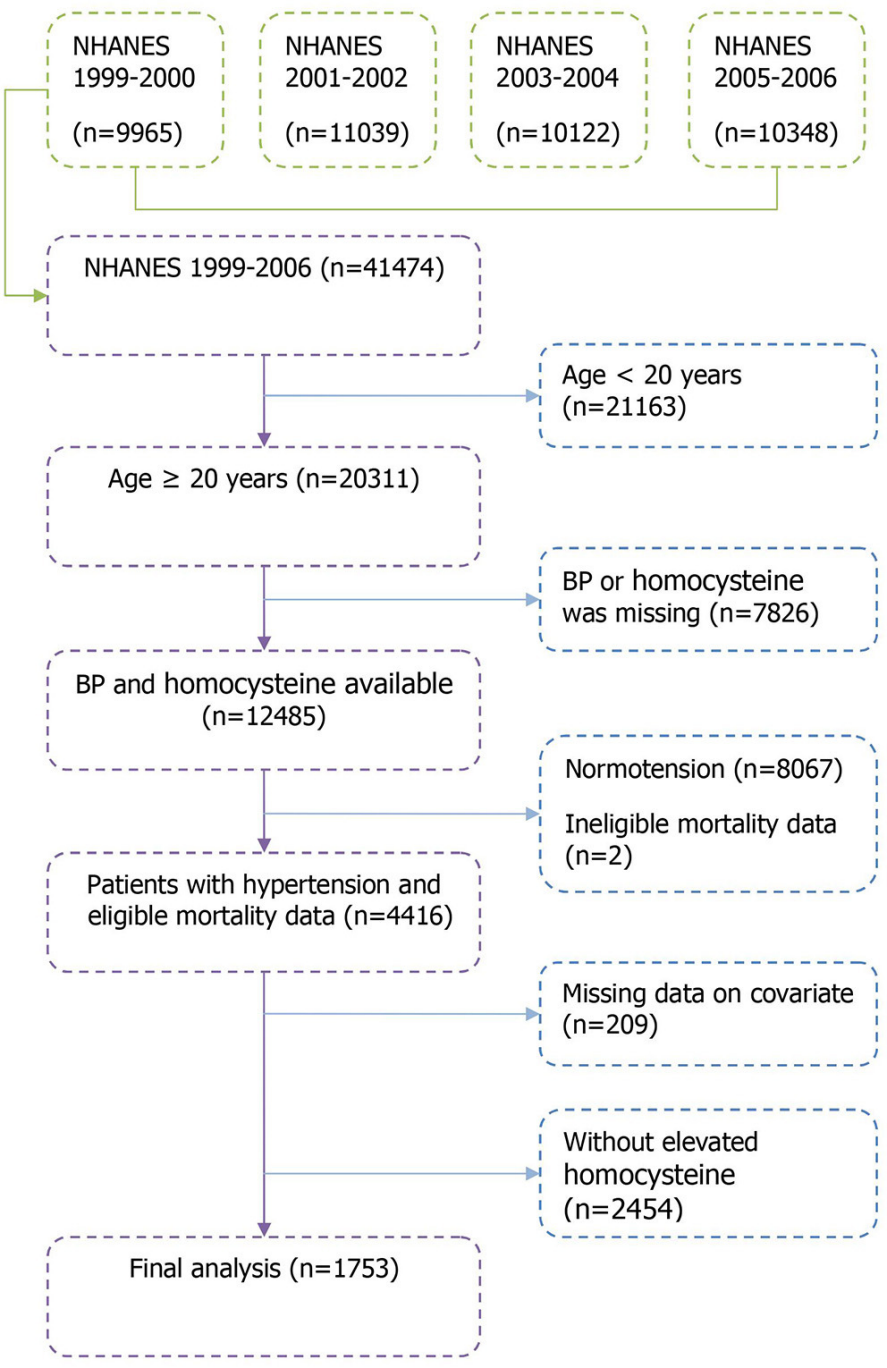
Flowchart of the study population selection.

Data were derived from 4 cycles of the NHANES (1999–2000, 2001–2002, 2003–2004, and 2005–2006) surveys, which were administered by the National Center for Health Statistics (NCHS). A detailed description of the NHANES study design and methods are available elsewhere (13). Briefly, NHANES is an ongoing health-related program conducted with a complex, multistage, probability sampling design for a representative sample of US residents. For this study, we merged the NHANES 1999–2006 with the 2015 NCHS Linked Mortality Files. In total, 41,474 individuals completed the NHANES surveys between 1999–2006 within 4 cycles (9,965 in the NHANES 1,999–2,000, 11,039 in the NHANES 2001–2002, 10,122 in the NHANES 2003–2004, and 10,348 in the NHANES 2005–2006). The present study was limited to hypertensive patients with elevated homocysteine aged ≥20 years with complete data on mortality and covariates, resulting in a sample of 1,753 participants in the analysis. A detailed flowchart depicting participant selection is shown in Figure 1. The NCHS institutional review board approved the study protocol and all participants provided written informed consent.

### Data Collection

Data were collected through interviews, medical examination and subsequent laboratory assessments in the Mobile Examination Centre. The NHANES interviews were completed by trained interviewers in participants' homes by using a computer-assisted personal interview system.

Demographics, health conditions, and lifestyle information were obtained through questionnaires. Smoking status was categorized into current, former and never smoker according to participants' answers. Drinking status includes drinkers and non-drinkers, where participants who drank alcohol at least 12 times in the previous year were defined as drinkers.

Bodyweight (kg) was measured using a digital weighing scale, and standing height (cm) was measured using a stadiometer. Body mass index (BMI) was calculated using the formula: weight (kg)/[height (m)]^2^. BMI ≥25 kg/m^2^ was defined as overweight/obese (14). For BP measurement, participants were asked to sit in a height-adjustable office-style chair and rest quietly for at least 5 min before taking BP readings. The mean of three systolic and diastolic BP readings was used in the analysis. Hypertension was defined as a systolic BP ≥140 mmHg or a diastolic BP ≥90 mmHg, and/or current use of antihypertensive medication.

### Laboratory Tests and Clinical Definitions

Serum specimens were processed, stored, and shipped to the Division of Environmental Health Laboratory Sciences, National Center for Environmental Health, Centers for Disease Control and Prevention for analysis. Both serum folate and vitamin B_12_ were measured by using the Bio-Rad Laboratories “Quantaphase II Folate/Vitamin B_12_” radioassay kit. RBC folate was calculated from the whole blood folate concentration using microbiologic assay by adjusting for RBC volume and correcting for serum total folate concentration, which was calculated as the sum of individual folate forms. The formula to calculate RBC folate is as follows:


$$\begin{array}{l} Red\;blood\;cell\;folate\;=\;\{whole\;blood\;folate-[serum\;blood\\ \;folate^{*}\;(1.0-hematocrit/100)]\}/(hematocrit/100) \end{array}$$

Hemoglobin A1c (HbA1c) was measured by high-performance liquid chromatography (HPLC), and we defined diabetes as HbA1c ≥6.5% and/or current treatment with a hypoglycemic agent or insulin. The laboratory method used for total cholesterol (TC) measurement was enzymatic assay. We defined hypercholesterolemia as the presence of TC ≥6.2 mmol/L or current medication use. C-reactive protein (CRP) was measured by latex-enhanced nephelometry on a Behring Nephelometer. The Chronic Kidney Disease Epidemiology Collaboration equation was used to estimate glomerular filtration rate (eGFR, in mL/min/1.73 m^2^) (15). Total homocysteine in plasma was measured by the Abbott Homocysteine assay on the Abbott AxSym analyzer, a fully automated fluorescence polarization immunoassay (FPIA) method.

### Ascertainment of Mortality Outcomes

Mortality information was extracted from the 2015 NCHS Public-Use Linked Mortality Files, including mortality status, cause of death, and follow-up time of all included participants from the date of survey participation until death or December 31, 2015, whichever was earlier. The leading cause of death falls into 9 cause-specific death categories based on the International Classification of Diseases, 10th Revision (ICD-10) code on the participant's death certificate (16). We defined all-cause mortality as death because of any reason, while cardiovascular mortality included deaths reportedly due to disease of the heart (leading death code 001) or cerebrovascular diseases (leading death code 005) (17).

### Statistical Analysis

Data were presented as mean ± standard deviation (SD) for continuous variables and as frequency (percentage) for categorical variables. RBC folate level was used in the analysis both as a categorical (quartiles) and a continuous (log-transformed) variable. We tested differences in baseline characteristics among folate quartile groups with one-way analysis of variance for continuous variables and χ2 tests for categorical variables. Crude all-cause and cardiovascular mortality rates by baseline serum and RBC folate quartiles were analyzed with the Kaplan-Meier curves and compared with the log-rank test. We then used Cox proportional hazards models to determine the hazard ratios (HRs) and 95% confidence intervals (CIs) for the risks of all-cause and cardiovascular mortality in each serum and RBC folate quartiles, using the lowest quartile as the reference. The models were adjusted for several potential confounding variables including age, sex, ethnicity, education, smoking status, drinking, overweight/obesity, diabetes, hypercholesterolemia, antihypertensive medication use, eGFR, serum vitamin B_12_, and CRP. To test for trend, the median value of each serum and RBC folate quartiles were used as continuous variables in the models. A two-tailed *p* value < 0.05 was considered statistically significant.

## Results

A total of 1,753 hypertensive patients with elevated homocysteine (serum homocysteine ≥10 μmol/L) were included in the analysis. The mean (SD) age was 68.5 (13.1) years at baseline. The baseline characteristics of the study sample by serum and RBC folate quartiles are shown in Tables 1, 2. Overall, patients who had higher folate levels were more likely to be older, women, white, of higher education levels and to have hypercholesterolemia. Besides, patients with higher folate levels were more likely to have higher serum vitamin B_12_ and lower eGFR levels.

**Table 1:** Baseline characteristics of hypertensive patients with elevated homocysteine by quartiles of serum folate.

**Characteristics**	**Quartile 1**	**Quartile 2**	**Quartile 3**	**Quartile 4**	***P*-values**
*N*	429	447	434	443	
Age, y	61.8 ± 14.4	67.7 ± 12.5	69.8 ± 12.1	74.5 ± 9.6	<0.0001
Men, No. (%)	286 (66.7)	256 (57.3)	255 (58.8)	227 (51.2)	<0.0001
Ethnicity, No. (%)					<0.0001
Hispanic	89 (20.8)	95 (21.3)	84 (19.4)	55 (12.4)	
White	176 (41.0)	219 (49.0)	263 (60.6)	331 (74.7)	
Black	148 (34.5)	119 (26.6)	78 (18.0)	45 (10.2)	
Asian or others	16 (3.7)	14 (3.1)	9 (2.1)	12 (2.7)	
Education, No. (%)					0.0046
Less than high school	182 (42.4)	204 (45.6)	175 (40.3)	142 (32.1)	
High school	109 (25.4)	107 (23.9)	118 (27.2)	134 (30.3)	
More than high school	138 (32.2)	136 (30.4)	141 (32.5)	167 (37.7)	
Smoking, No. (%)					<0.0001
Never	159 (37.1)	195 (43.6)	195 (44.9)	210 (47.4)	
Current	130 (30.3)	88 (19.7)	65 (15.0)	27 (6.1)	
Former	140 (32.6)	164 (36.7)	174 (40.1)	206 (46.5)	
Drinker, No. (%)	279 (65.0)	274 (61.3)	276 (63.6)	254 (57.3)	0.0988
Overweight/obese, No. (%)	340 (79.3)	345 (77.2)	324 (74.7)	315 (71.1)	0.0328
Diabetes, No. (%)	69 (16.1)	124 (27.7)	98 (22.6)	100 (22.6)	0.0006
Hypercholesterolemia, No. (%)	153 (35.7)	197 (44.1)	187 (43.1)	222 (50.1)	0.0003
Antihypertensive medication use, No. (%)	268 (62.5)	321 (71.8)	310 (71.4)	342 (77.2)	<0.0001
eGFR, mL/min/1.73 m^2^	77.3 ± 23.8	70.3 ± 23.2	68.9 ± 21.8	59.3 ± 22.6	<0.0001
Log-transformed serum vitamin B_12_, pg/mL	5.9 ± 0.5	5.9 ± 0.5	6.0 ± 0.5	6.2 ± 0.6	<0.0001
Log-transformed CRP, mg/dL	−1.1 ± 1.2	−1.2 ± 1.2	−1.4 ± 1.1	−1.3 ± 1.1	0.0009

*CRP, C-reactive protein; eGFR, estimated glomerular filtration rate. The cut-off values of serum folate were 8.3, 12.0, and 18.4 ng/mL*.

**Table 2:** Baseline characteristics of hypertensive patients with elevated homocysteine by quartiles of red blood cell folate.

**Characteristics**	**Quartile 1**	**Quartile 2**	**Quartile 3**	**Quartile 4**	***P*-values**
*N*	433	439	439	442	
Age, y	65.6 ± 14.1	67.1 ± 13.1	68.9 ± 13.2	72.3 ± 10.8	<0.0001
Men, No. (%)	260 (60.1)	274 (62.4)	262 (59.7)	228 (51.6)	0.0069
Ethnicity, No. (%)					<0.0001
Hispanic	82 (18.9)	102 (23.2)	80 (18.2)	59 (13.4)	
White	160 (37.0)	217 (49.4)	292 (66.5)	320 (72.4)	
Black	171 (39.5)	108 (24.6)	57 (13.0)	54 (12.2)	
Asian or others	20 (4.6)	12 (2.7)	10 (2.3)	9 (2.0)	
Education, No. (%)					0.0004
Less than high school	213 (49.2)	178 (40.6)	162 (36.9)	150 (33.9)	
High school	99 (22.9)	112 (25.5)	129 (29.4)	128 (29.0)	
More than high school	121 (27.9)	149 (33.9)	148 (33.7)	164 (37.1)	
Smoking, No. (%)					<0.0001
Never	170 (39.3)	191 (43.5)	201 (45.8)	197 (44.6)	
Current	123 (28.4)	92 (21.0)	60 (13.7)	35 (7.9)	
Former	140 (32.3)	156 (35.5)	178 (40.6)	210 (47.5)	
Drinker, No. (%)	260 (60.1)	288 (65.6)	281 (64.0)	254 (57.5)	0.0532
Overweight/obese, No. (%)	318 (73.4)	332 (75.6)	351 (80.0)	323 (73.1)	0.0684
Diabetes, No. (%)	74 (17.1)	112 (25.5)	87 (19.8)	118 (26.7)	0.0012
Hypercholesterolemia, No. (%)	167 (38.6)	173 (39.4)	204 (46.5)	215 (48.6)	0.0035
Antihypertensive medication use, No. (%)	273 (63.1)	310 (70.6)	311 (70.8)	347 (78.5)	<0.0001
eGFR, mL/min/1.73 m^2^	76.1 ± 24.6	72.4 ± 21.7	68.4 ± 21.2	58.9 ± 23.8	<0.0001
Log-transformed serum vitamin B_12_, pg/mL	5.9 ± 0.5	5.9 ± 0.5	6.0 ± 0.5	6.3 ± 0.5	<0.0001
Log-transformed CRP, mg/dL	−1.4 ± 1.2	−1.3 ± 1.1	−1.3 ± 1.2	−1.2 ± 1.1	0.0209

*CRP, C-reactive protein; eGFR, estimated glomerular filtration rate. The cut-off values of RBC folate were 209, 278, and 381 ng/mL*.

**Figure 2:**
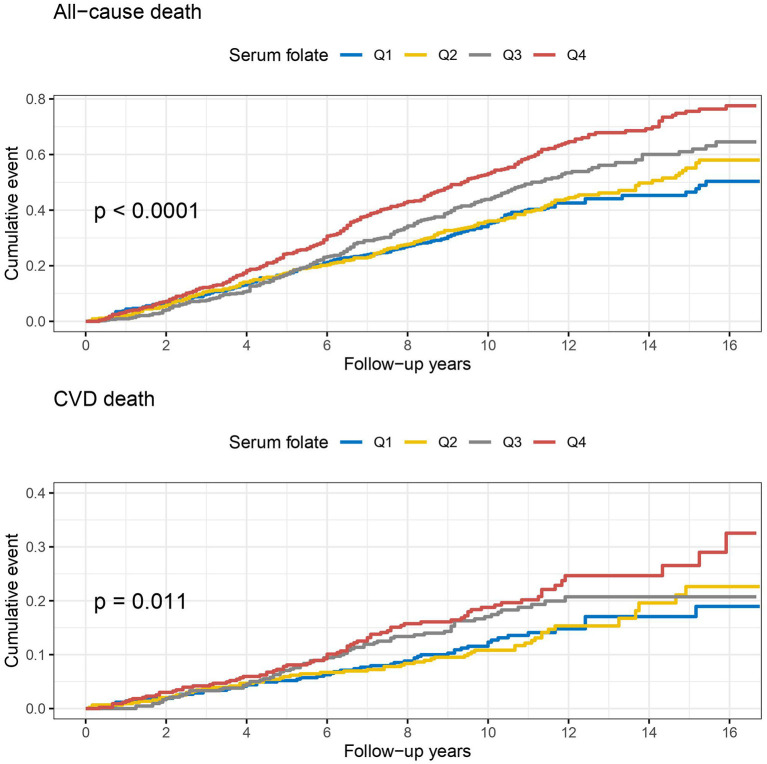
Kaplan-Meier curves of all-cause and cardiovascular disease (CVD) death by baseline quartiles of serum folate.

**Figure 3:**
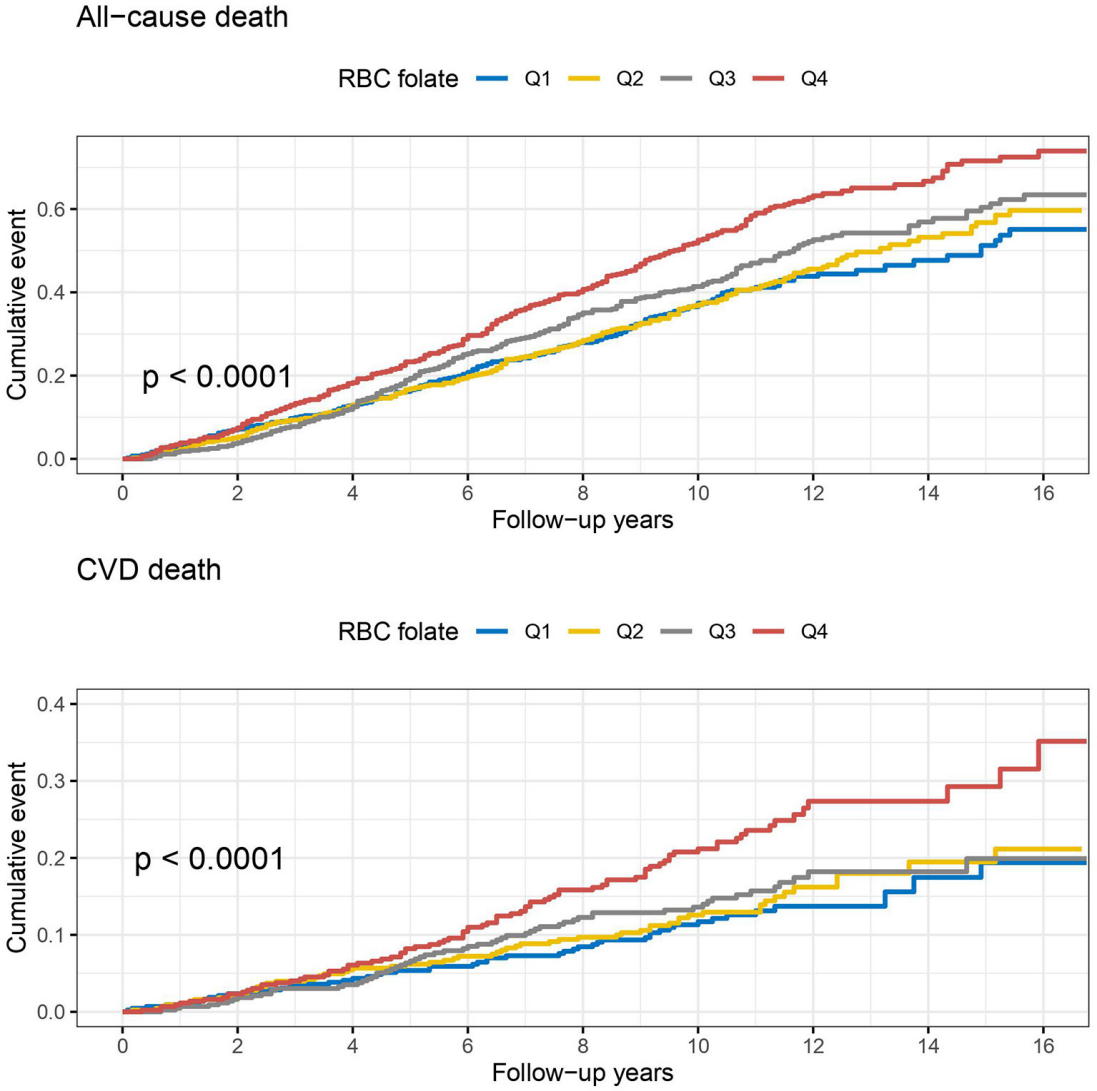
Kaplan-Meier curves of all-cause and cardiovascular disease (CVD) death by baseline quartiles of red blood cell (RBC) folate.

During a median follow-up of 10.0 years, a total of 899 all-cause deaths including 257 CVD deaths occurred among the 1,753 hypertensive patients with elevated homocysteine. The Kaplan-Meier curves indicate a graded increased risk of all-cause and CVD mortality associated with higher serum and RBC folate concentrations (log-rank *p* < 0.05) (Figures 2, 3).

The unadjusted and adjusted HRs and 95% CIs of serum and RBC folate for all-cause and CVD mortality are shown in Table 3. Comparison of the highest with the lowest quartile of serum folate showed a higher risk of all-cause (HR: 1.83, 95% CI: 1.52–2.20) and CVD (HR: 1.61, 95% CI: 1.14–2.29) mortality in the unadjusted model. However, the associations were no longer significant after adjustment for age, sex, ethnicity, education, smoking status, drinking, overweight/obesity, diabetes, hypercholesterolemia, antihypertensive medication use, eGFR, serum vitamin B_12_, and CRP. For RBC folate, the unadjusted HRs and 95% CIs comparing the highest to lowest quartile were 1.64 (1.36–1.97) for all-cause death and 1.91 (1.35–2.70) for CVD death. After further adjustments for covariates, the HRs and 95% CIs were 1.13 (0.92–1.39) for all-cause death and 1.47 (1.01–2.16) for CVD death. There was a significant and positive trend between RBC folate and the risk of CVD death among hypertensive patients with elevated homocysteine (p for trend = 0.0196).

**Table 3:** Mortality risk among hypertensive patients with elevated homocysteine by quartiles of serum and red blood cell folate.

	**Quartile 1**	**Quartile 2**	**Quartile 3**	**Quartile 4**	**P for trend**
**Serum folate**					
Person-years of follow-up	4,066	4,294	4,001	3,777	
**All-cause mortality**					
No. with events	177	200	230	292	
Mortality rate, per 1,000 person-years	43.5	46.6	57.5	77.3	
Unadjusted HR (95% CI)	1 (Reference)	1.07 (0.87–1.31)	1.34 (1.10–1.63)	1.83 (1.52–2.20)	<0.0001
Adjusted HR (95% CI)^a^	1 (Reference)	0.75 (0.61–0.92)	0.85 (0.69–1.04)	0.91 (0.74–1.12)	0.5691
Adjusted HR (95% CI)^a^ of per 1-unit increase of log-transformed folate	1.03 (0.91–1.16)				0.6457
**Cardiovascular mortality**					
No. with events	53	56	70	78	
Mortality rate, per 1,000 person-years	13.0	13.0	17.5	20.7	
Unadjusted HR (95% CI)	1 (Reference)	1.00 (0.69–1.46)	1.36 (0.95–1.94)	1.61 (1.14–2.29)	0.0016
Adjusted HR (95% CI)^a^	1 (Reference)	0.69 (0.47–1.01)	0.87 (0.60–1.27)	0.84 (0.57–1.24)	0.9906
Adjusted HR (95% CI)^a^ of per 1-unit increase of log-transformed folate	1.02 (0.82-1.27)				0.8518
**Red blood cell folate**					
Person-years of follow-up	4,073	4,210	4,083	3,771	
**All-cause mortality**					
No. with events	188	204	229	278	
Mortality rate, per 1,000 person-years	46.2	48.5	56.1	73.7	
Unadjusted HR (95% CI)	1 (Reference)	1.05 (0.86–1.28)	1.22 (1.01–1.48)	1.64 (1.36–1.97)	<0.0001
Adjusted HR (95% CI)^a^	1 (Reference)	0.94 (0.77–1.15)	1.01 (0.82–1.24)	1.13 (0.92–1.39)	0.0909
Adjusted HR (95% CI)^a^ of per 1-unit increase of log-transformed folate	1.16 (0.99–1.36)				0.0670
**Cardiovascular mortality**					
No. with events	50	59	61	87	
Mortality rate, per 1,000 person-years	12.3	14.0	14.9	23.1	
Unadjusted HR (95% CI)	1 (Reference)	1.14 (0.78–1.66)	1.22 (0.84–1.77)	1.91 (1.35–2.70)	<0.0001
Adjusted HR (95% CI)^a^	1 (Reference)	1.02 (0.70–1.51)	1.07 (0.72–1.58)	1.47 (1.01–2.16)	0.0196
Adjusted HR (95% CI)^a^ of per 1-unit increase of log-transformed folate	1.36 (1.01–1.82)				0.0412

a *Adjusted for age, sex, ethnicity, education, smoking, drinking, overweight/obese, diabetes, hypercholesterolemia, antihypertensive medication use, estimated glomerular filtration rate, serum vitamin B_12_, and C-reactive protein. HR, hazard ratio; CI, confidence interval*.

## Discussion

In this prospective cohort study of a nationally representative sample of US adults, we found that RBC folate was positively associated with the risk of cardiovascular mortality among patients with both hypertension and elevated homocysteine, which is referred to as H-type hypertension. This association was independent of multiple potential covariates including demographics, lifestyles, cardiometabolic risk factors, renal function, and others. However, no significant association was found between serum folate and mortality after adjusting for potential covariates.

Although the associations between folate levels and mortality have been previously investigated, the current evidence is not yet conclusive: some studies did not find any significant associations (18, 19); among studies that did, J- or U-shaped associations (20–23), positive linear associations (24), and inverse associations (17, 25, 26) have all been reported; additionally, two studies found that only low folate levels were significantly associated with increased risk of mortality (27, 28). However, most of the previous studies used serum folate as the main exposure, and only two studies used RBC folate in the analysis (21, 24). This might be an explanation for the conflicting results, as serum folate only reflects recent intakes while RBC folate reflects long-term average consumption of folate (12).

To our knowledge, no previous studies specifically explored the long-term effects of serum and RBC folate on the risk of mortality among hypertensive patients with elevated homocysteine. Folate plays an important role in the metabolism of homocysteine by serving as the methyl donor in the conversion of homocysteine to methionine (29). Folic acid supplementation has been observed to be effective in reducing plasma homocysteine levels (30). Folic acid therapy has been recommended to patients with “H-type hypertension” to achieve potential cardiovascular benefits (7, 10). Our previous analysis found that the RBC folate levels in folic acid supplement users were much higher than those in non-users (mean RBC folate concentration: 728 vs. 494 ng/mL) (11). The current study suggests that long-term cardiovascular death risk should be assessed properly when folic acid therapy is considered for patients diagnosed with H-type hypertension.

Considering that older people are at greater risk of death than younger adults. In addition to adjusting for age, we also performed stratified analysis according to age (≤ 65 or >65 years). The adjusted HRs of per 1-unit increase of log-transformed RBC folate for CVD death in younger and older adults were 1.24 (0.62–2.44) and 1.43 (1.03–1.99), respectively. However, no significant interaction was found between RBC folate and age (p for interaction = 0.4592), which means that the relationship between RBC folate level and CVD death in different age groups cannot be considered to have essential difference.

The mechanism underlying the association between high RBC folate and increased risk of CVD mortality among hypertensive patients with elevated homocysteine remains unclear. One possible explanation is homocysteine-induced arterial calcification and atherosclerosis (31). As mentioned above, although folate serves as the methyl donor in the conversion of homocysteine to methionine, high folate status may decrease the availability of the methyl donor or impair the activity of methyl-THF reductase, which is responsible for the methylation of homocysteine (32). Consequently, homocysteine accumulates, leading to arterial calcification and atherosclerosis. High levels of RBC folate were associated with a high risk of severe abdominal aortic calcification, which might be evidence for this theory (11). In contrast, we did not find a significant association between serum folate and mortality among hypertensive patients with elevated homocysteine, which suggests that serum and RBC folate play different roles in the etiology of certain diseases. Routinely monitoring of RBC folate is recommended for folic acid supplement users.

This study has several limitations. First, although the NHANES population is a nationally representative sample of US adults, we limited the sample of this study to hypertensive patients, which may impair the representativeness. Second, limited by the small sample size, we only included CVD-related mortality but not other cause-specific mortality. Third, due to the lack of data on folic acid supplement use, we were unable to further differentiate the associations between RBC folate and mortality by folic acid supplement use history. Fourth, folate status was investigated only once at baseline. Patients' lifestyle habits including diet and tobacco smoking likely changes after diagnosis of H-type hypertension. Such changes could have an impact on long-term outcome. Unfortunately, this study did not collect these mentioned variables during the follow-up, and this limitation needs to be improved in future studies.

## Conclusions

In conclusion, findings from our study suggest that a high level of RBC folate is associated with an increased risk of cardiovascular mortality among hypertensive patients with elevated homocysteine. Further studies on larger samples are warranted to confirm our findings.

## Data Availability Statement

The raw data supporting the conclusions of this article will be made available by the authors, without undue reservation.

## Ethics Statement

The studies involving human participants were reviewed and approved by National Center for Health Statistics (NCHS). The patients/participants provided their written informed consent to participate in this study.

## Author Contributions

LZhou designed the present study and performed data analysis. LZhou and HH contributed equally to the writing of this article. XW, LZhao, ML, JT, and GL critically revised and edited the manuscript for important intellectual content. YC, JL, FC, and GL contributed to the data interpretation. All authors approved the final version of the manuscript to be published.

## Funding

This work was supported by the Scientific Research Starting Funding for Young Scholars in Sichuan Provincial People's Hospital. The funding source had no role in the design, analysis, or writing of this manuscript.

## Conflict of Interest

The authors declare that the research was conducted in the absence of any commercial or financial relationships that could be construed as a potential conflict of interest.

## Publisher's Note

All claims expressed in this article are solely those of the authors and do not necessarily represent those of their affiliated organizations, or those of the publisher, the editors and the reviewers. Any product that may be evaluated in this article, or claim that may be made by its manufacturer, is not guaranteed or endorsed by the publisher.
